# Mechanical Parameters and Trajectory of Two Chinese Cervical Manipulations Compared by a Motion Capture System

**DOI:** 10.3389/fbioe.2021.714292

**Published:** 2021-07-26

**Authors:** Xuecheng Huang, Dongxin Lin, Zeyu Liang, Yuping Deng, Zaopeng He, Mian Wang, Jinchuan Tan, Yikai Li, Yang Yang, Wenhua Huang

**Affiliations:** ^1^National Key Discipline of Human Anatomy, School of Basic Medical Sciences, Southern Medical University, Guangzhou, China; ^2^Guangdong Engineering Research Center for Translation of Medical 3D Printing Application, Southern Medical University, Guangzhou, China; ^3^Guangdong Provincial Key Laboratory of Medical Biomechanics, Southern Medical University, Guangzhou, China; ^4^Shenzhen Hospital of Guangzhou University of Chinese Medicine, Shenzhen, China; ^5^Hand and Foot Surgery and Plastic Surgery, Affiliated Shunde Hospital of Guangzhou Medical University, Foshan, China; ^6^School of Chinese Medicine, Southern Medical University, Guangzhou, China

**Keywords:** motion capture, cervical spondylotic radiculopathy, cervical rotation manipulation, thrust range of motion, active range of motion, passive range of motion

## Abstract

**Objective:** To compare the mechanical parameters and trajectory while operating the oblique pulling manipulation and the cervical rotation–traction manipulation.

**Methods:** An experimental research measuring kinematics parameter and recording motion trajectories of two cervical manipulations were carried out. A total of 48 healthy volunteers participated in this study, who were randomly divided into two groups of 24 representing each of the two manipulations. A clinician performed two manipulations in two groups separately. A motion capture system was used to monitor and analyze kinematics parameters during the operation.

**Results:** The two cervical manipulations have similar thrust time, displacement, mean velocity, max velocity, and max acceleration. There were no significant differences in active and passive amplitudes between the two cervical rotation manipulations. The thrust amplitudes of the oblique pulling manipulation and the cervical rotation–traction manipulation were 5.735 ± 3.041° and 2.142 ± 1.742°, respectively. The thrust amplitudes of the oblique pulling manipulation was significantly greater than that of the cervical rotation–traction manipulation (*P* < 0.001).

**Conclusion:** Compared with the oblique pulling manipulation, the cervical rotation–traction manipulation has a less thrust amplitudes.

## Introduction

Cervical spinal manipulation is proven to be effective in improving the range of motion of the cervical spine and relieve pain (Bronfort et al., 2004; Vernon and Humphreys, 2008), largely due to its high-speed and low-amplitude (HVLA) operating characteristics (Galindez-Ibarbengoetxea et al., 2017; Gómez et al., 2020). HVLA techniques can be defined as it uses low amplitude, high speed thrusts where the vertebrae are taken out of their normal physiological range of motion without surpassing the boundary of anatomical integrity (Giacalone et al., 2020). HVLA techniques has a positive effect on reducing neck pain (Ruiz-Sáez et al., 2007), increasing cervical spine mobility (Martínez-Segura et al., 2006), and improving posture (Smith and Mehta, 2008) by acting on the facet joints and soft tissues including muscles and ligaments. However, manipulations are greatly diverse, and lack of diagnoses, therapeutic standards, and complete evaluation systems which are used for the mechanical parameters and safety indexes. This dilemma limits the development and communication of CSM and predisposes to serious complications of the various structures involved in cervical spine injury, mainly including soft tissue injury, aggravation of disk herniation, and even spinal cord injury (Gorrell et al., 2016; Nielsen et al., 2017; Kranenburg et al., 2017a,b).

Therefore, it is of considerable importance to determine the key biomechanical parameters of the operation (Herzog, 2010). Cervical rotation manipulation (CRM) is one of the cervical spine manipulation techniques, which has a long history and is widely used in China (Zhu et al., 2005). The oblique pulling manipulation (Zhang and Gu, 2014) and the rotation–traction manipulation (Huang et al., 2017) both are CRM, and they are commonly used operations in clinical practice (Anderst et al., 2018). From a kinematic point of view, the process of any CRMs involve first flexing and rotating the cervical spine to a specific angle and then applying a rotational force that causes slight displacement of adjacent tissues, such as vertebrae and disks in the space. Here, “force, direction, angle, speed, displacement, and time” constitute the essence of the manipulation effect. Consequently, quantifying the operational characteristics of the CRM will help to standardize it and indirectly ensure its therapeutic effect (Triano et al., 2003; Needham et al., 2016).

Motion capture is currently widely used in the animation industry and sport medical biomechanics (Menolotto et al., 2020). Optical systems have been considered the gold standard for motion capture in the literature (van der Kruk and Reijne, 2018). It is a precision device for accurately measuring, capturing, and recording the motion of moving objects in a spatial coordinate system that has emerged in the last decade. It can be used to extract and analyze trajectories and characteristics of movements during an operation, resulting in very accurate measurements of technical specifications that can guide clinicians and serve as the basis for mechanistic studies (Boser et al., 2018). When the motion capture object is a real person, the marker is typically a human anatomical bone process or joint, and the corresponding model and identification is localized (Liu et al., 2020). Motion capture devices can track and record motion data for each marker including the trajectory, speed, acceleration, and angle of each joint of the body. Therefore, we take the unique advantage of the optical motion capture system to compare the mechanical parameters and trajectories when operating the oblique pulling manipulation and the cervical rotation-traction manipulation.

## Materials and Methods

A total of 48 volunteers (20 women and 28 men) aged from 24 to 30 years old, who had no pathological changes after X-ray examination, were selected. They were randomly divided into the group of the oblique pulling manipulation and the group of the rotation–traction manipulation. A total of 24 subjects were in each group. A senior clinician was involved in the study. Before the experiment, the volunteers were massaged on the neck for 5–10 min to relax and were informed and familiar with the entire experimental process. All subjects signed informed consent before participation, and the project was approved by the Medical Ethics Committee of Shunde Hospital, Guangzhou Medical University.

### Instrumentation

The digital motion capture system was composed of 12 sets of infrared motion capture cameras (model: Miqus M3, origin: Qualisys, Sweden; full view standard model: 2 million pixels, 340 FPS sampling rate; full view model: 500,000 pixels, 650 FPS sampling rate; 0.1 mm accuracy), which were placed throughout the room to collect the kinematics data. Visual 3D software (origin: C-Motion) was used to analyze and rebuild the three-dimensional images.

### Procedures

#### Field Calibration

This study was implemented at the Southern Medical University of Basic Medicine. First, an L-shaped calibrator (including three points and two gradienters) was used to perform static calibration for the horizontal plane and the origin of coordinates. Then, a T-shaped calibrator (comprising three points) was used to perform the dynamic calibration by waving the calibrator constantly in the experimental site. The three-dimensional space Cartesian coordinate system was defined through the calibration of the horizontal, coordinate, and origin space. The *X*-axis represents the frontal axis, the *Y*-axis represents the sagittal axis, and the *Z*-axis represents the vertical axis.

#### Marker Fixation

**FIGURE 1:**
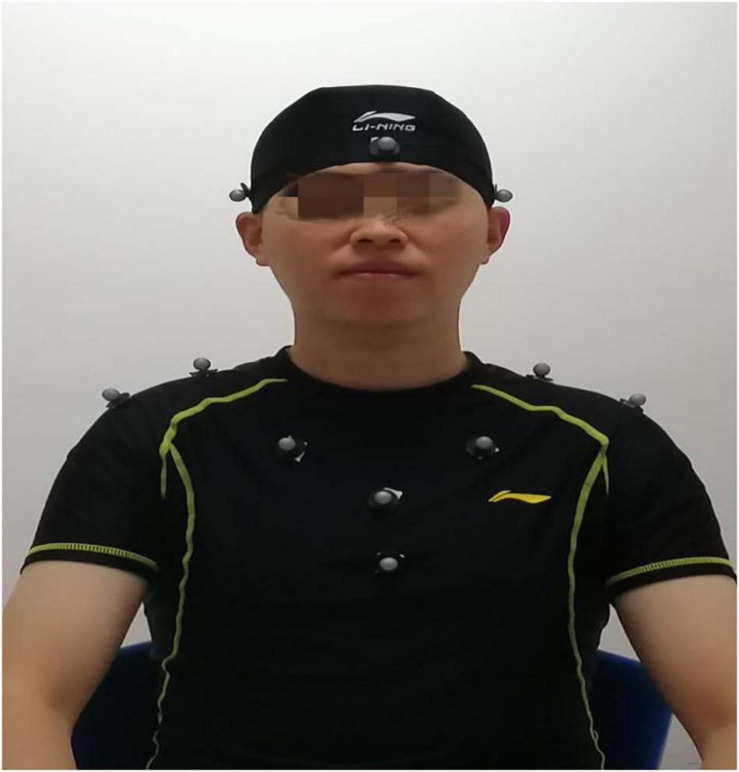
Position of marker points on the volunteers.

**FIGURE 2:**
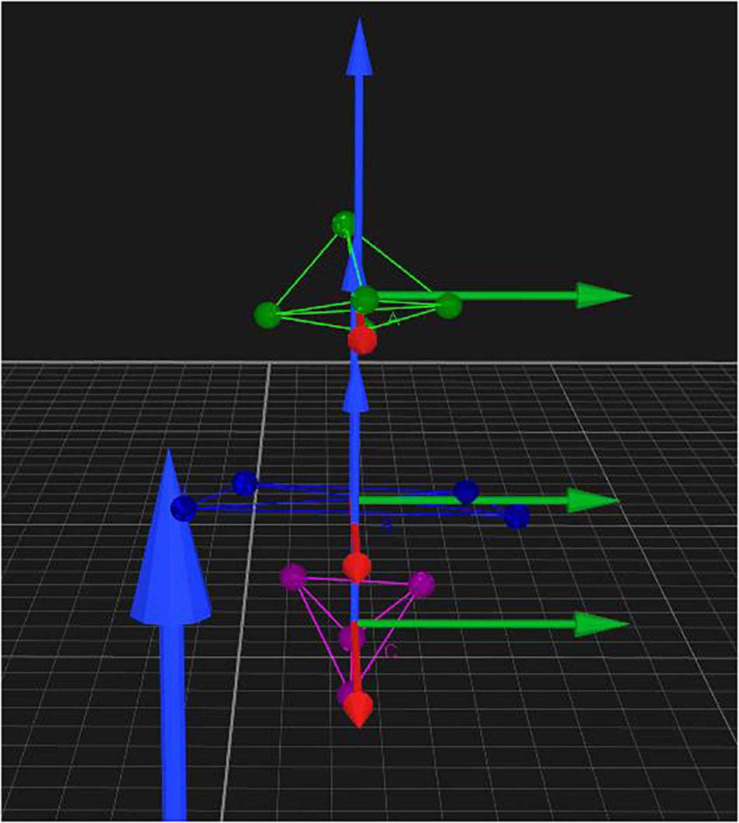
Position of markers displayed in the motion capture system and the relationship and coordinate system between the head rigid body and the torso rigid body.

After volunteers put on straitjackets and caps, 13 special marker points were placed in the head and trunk to establish three-dimensional models. The specific positions were as follows (as shown in Figure 1): five marker points were on the head (one point each on the bilateral temporal regions, one point on the forehead, one point on the vertex, and one point on the occipital region), four points on the shoulder and neck (one point each on the bilateral acromions and one point each on the midline bilateral clavicles), and a four points on the trunk (one point each on the bilateral pectoralis major muscles, one point under the xiphoid, and one point on the upper abdomen). The location of the marker points displayed in the motion capture system and the relationship and coordinate system between the head rigid body and the torso rigid body is shown in Figure 2.

#### Formal Experiment

**FIGURE 3:**
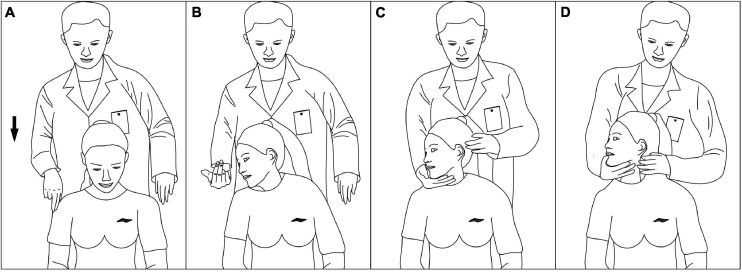
Process of the oblique pulling manipulation. **(A)** Flex. **(B)** Active rotation. **(C)** Passive rotation. **(D)** Instant pull.

**FIGURE 4:**
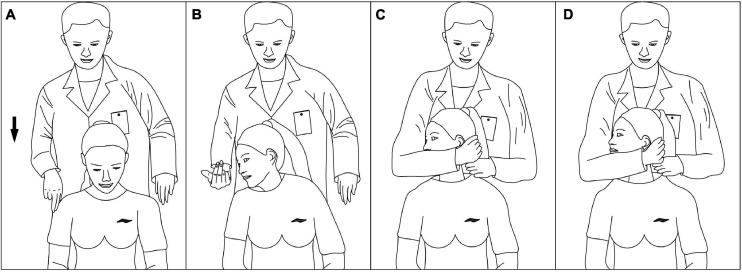
Process of the cervical rotation–traction manipulation. **(A)** Flex. **(B)** Rotary position. **(C)** Pretraction. **(D)** Upward-thrust.

Before the experiment, the first volunteer stood on the spot, arms outstretched for system calibration. The volunteers were manipulated in a upright seated position, and the clinician stood behind the volunteers. The two cervical spine manipulations we are comparing are non-fixed-point rotational manipulations. They are not performed on a specific cervical segment, but use the wrenching of the head to transmit force to the cervical spine to achieve a therapeutic effect. The clinician performs the oblique pulling and the rotation-traction manipulation on the left and right sides of the subjects by group. The oblique pulling manipulation was based on the manipulation rules formulated by Yao et al. (2012). Take the oblique pulling manipulation on the right as an example (shown in Figure 3). The specific steps were as follows. (A) Flexion: guide the subject’s head into flexion. (B) Active Rotation: extreme rotation in the right direction. (C) Passive rotation: the practitioner uses the right elbow against the volunteer’s left shoulder, the right hand against the back of the volunteer’s neck, and the left hand against the volunteer’s jaw to help the subject bend again to the right limit. (D) Transient pull: the clinician momentarily increases the force of rotation and then releases it. The oblique pulling manipulation is done with one hand against the participant’s jawbone and the other hand against the neck, with the two hands exerting concerted force in opposite directions, allowing the neck to be mildly twisted to the point of apparent resistance. The rotation–traction manipulation was based on the manipulation rules formulated by Liguo et al. (2017). The right side of the rotation–traction manipulation is used as an example (shown in Figure 4). The specific steps were as follows. (A) Flex: the volunteer’s head was guided to flex. (B) Rotary position: the head was rotated to the right direction limit, then the clinician helped the subjector to rotate to the right direction limit again. (C) Pretraction: the volunteer’s mandible was held in the clinician’s forearm and then pulled slowly upward for approximately 3–5 s. (D) Upward-thrust: the head was thrust upward rapidly after pretraction. The cervical rotation-traction manipulation is to use the clinician’s elbow against the participant’s jawbone, the other hand against the neck, and the elbow with a short force to quickly thrust upward. The manipulation procedure was captured dynamically by the motion capture system.

### Data Analysis and Post-Processing

The saved data were analyzed and processed by Visual 3D analysis software. According to the anatomical features, the five points of the head are used as the rigid body of the head, and the four points of the shoulders and the four points of the upper abdomen are used as the rigid body of the trunk. The relative motion between the head rigid body and the trunk rigid body was calculated. We used the mean values of the data obtained from the left and right sides. Finally, the thrust time, thrust displacement, mean thrust velocity, maximum thrust velocity, maximum thrust acceleration, thrust angular displacement, active motion amplitude, and passive motion amplitude of the 48 volunteers obtained were analyzed using the statistical package SPSS 19.0. A two independent samples *t*-test was used to compare two groups of data.

## Results

Baseline information for the two groups is compared in Table 1, and there were no differences in gender distribution, subject age, height, or weight between the two groups to allow for a subject study.

**TABLE 1:** Comparison of baseline information between the two groups.

	**Oblique pulling manipulation**	**Cervical rotation–tractionmanipulation**	***P***
Age (y)	27.30 ± 4.28	26.90 ± 3.27	0.882
Height (cm)	173.00 ± 7.63	172.90 ± 9.56	0.980
Weight (kg)	67.93 ± 11.65	65.30 ± 13.31	0.602
BMI	22.72 ± 2.36	22.06 ± 3.29	0.892

*BMI = Weight (kg)/Height (m^2^).*

The kinematic parameters of the participants are listed in Table 2. The thrust time, thrust displacement, average thrust speed, max thrust speed, and max thrust acceleration all showed no significant difference between two manipulations (*P* > 0.05).

**TABLE 2:** Comparison of kinematic parameters of participants between the two groups.

	**Oblique pulling manipulation**	**Cervical rotation–traction manipulation**	***P***
Thrust time (s)	0.240 ± 0.054	0.252 ± 0.041	0.188
Thrust displacement (mm)	35.19 ± 14.67	34.65 ± 12.40	0.833
Average thrust speed (mm/s)	150.89 ± 67.71	138.20 ± 47.50	0.251
Maximum thrust speed (mm/s)	216.12 ± 91.45	193.88 ± 65.12	0.139
Maximum thrust acceleration (mm/s^2^)	6399.49 ± 2115.26	5352.57 ± 3683.69	0.233

Table 3 presents the mean amplitude of active and passive motion during two manipulations. No significant difference is found between the oblique pulling manipulation and the cervical rotation–traction manipulation (*P* > 0.05).

**TABLE 3:** Comparion of active and passive motion amplitude between the two groups (°).

	**Oblique pulling manipulation**	**Cervical rotation–traction manipulation**	***P***
Active movement amplitude	67.18 ± 4.31	67.72 ± 3.83	0.485
Passive motion amplitude	77.74 ± 4.15	79.22 ± 4.16	0.059

The mean thrust angular displacement of two manipulations is shown in Table 4. The thrust amplitudes of the oblique pulling manipulation significantly greater than the cervical rotation–traction manipulation (*P* < 0.001).

**TABLE 4:** Comparison of thrust angular displacement between the two groups (°).

	**Oblique pulling manipulation**	**Cervical rotation–traction manipulation**	***P***
Thrust angular displacement	5.735 ± 3.041	2.142 ± 1.724	0.000

## Discussion

This study adopts three-dimensional motion capture technology to conduct kinematic analysis of the operational characteristics of the two techniques, so as to conduct a comprehensive analysis of the techniques from the perspective of three-dimensional structure; to conduct a detailed and thorough study of the techniques by measuring the frequency, velocity, acceleration and other data of the techniques; and to make a convincing comparison of the safety of the two techniques. Therefore, using numerical or mechanical language to describe the mechanical characteristics of the Chinese manipulation will help standardize the manipulation and indirectly ensure the efficacy of the manipulation.

In motion capture systems, marker points must be placed in such a way that they not only represent head and torso movements, but also that they are not obscured by the clinician (Price et al., 2020). The head and torso are marker points placed according to anatomical bony landmarks, where the five points on the head constitute the rigid body of the head, and the four points on the shoulders and the four points on the upper abdomen constitute the rigid body of the torso. The relative motion between the rigid body of the head and the rigid body of the trunk indirectly reflects the motion of the cervical spine.

Van Zoest and Gosselin (2003) directly measured the three-dimensional interactions between physician limbs and patients during manipulation and showed a significant advantage of the presence of three-dimensional mechanical parameters over unidirectional mechanical parameters. However, they did not study the thrust velocity and acceleration. In this study, we successfully captured the mechanical parameters and the trajectory of two cervical manipulations. Table 2 shows that the kinematic parameters of the two manipulations are more consistent when thrust. We observed that both manipulations meet the characteristics of HVLA cervical spine manipulation techniques by comparing with Zhu’s experimental results (van der Kruk and Reijne, 2018; thrust velocity: 203.06 ± 49.95 mm/s; thrust acceleration: 3836.27 ± 1262.28 mm/s^2^). Statistical analysis showed that the mechanical parameters of the two methods were not statistically significant. We confirmed that there were no significant differences in kinematic characteristics between the oblique pulling manipulation and the cervical rotation–traction manipulation.

Table 3 shows the active and passive motion amplitudes of the two groups. Active range of motion is when the volunteer actively flexes and rotates to the side to the limit. Passive range of motion is the volunteer first actively moves as far as possible and the clinical then passively continues the movement until the maximum passive ROM is reached. Both active and passive movements are within the range of physiological activity (Tousignant et al., 2006). The amplitude of active and passive motion for the oblique pulling manipulation was 67.18 ± 4.31° and 77.74 ± 4.15°, respectively. The amplitudes of cervical rotation–traction manipulation were 67.72 ± 3.83° and 79.22 ± 4.16°, respectively. The present experimental results are similar to the active and passive rotation values measured by Mei et al. (2010) using a laser scanner (global positioning system coordinates, accuracy 0.01°), at 68.37 ± 3.32° and 78.94 ± 4.46°. In addition, the present experiment is similar to the rotation angle data measured by Feipel et al. (1999) for the cervical spine in flexion, again validating the reliability of the data in this study. In summary, the amplitude of active and passive movements of the cervical spine is similar in both manipulations.

The thrust amplitude of the oblique pulling manipulation was more than twice of that the cervical rotation-traction manipulation as Table 4 shown.

### The Reason Why the Oblique Pulling Manipulation Has a Larger Thrust Amplitude Than the Cervical Rotation-Traction Manipulation

From the third and fourth pictures of Figures 3, 4, it can be seen that the pretraction position of the cervical rotation-traction manipulation does not differ much from the position of the thrust process, whereas the oblique pulling manipulation undergoes a large change in position before and after the thrust. We may be able to analyze the reasons for this result in terms of the manipulative characteristics of the two manipulations by the motion capture system. The oblique pulling manipulation is divided into three main processes: active rotation of the subject to the limit, fixed angle after the clinician helps the subject reach the limit again with passive movement, and sudden pulling. While the main steps of the cervical rotational-traction manipulation involves active rotation of the subject to the limit. The clinician then pretracted the subject forward to a fixed angle and abruptly thrusted upward. The oblique pulling manipulation is dominated by rotation during traction and upward lifting. And the cervical rotational traction method is mainly upward lifting with rotation as an aid during traction. The different manipulative characteristics of the two methods of thrusting are directly responsible for the different thrust amplitudes. Zhu (van der Kruk and Reijne, 2018) used 12 digital motion capture lenses to dynamically capture the operation process of the CRM. The results show that the force direction of thrust is mainly vertically upward. In the mean time, the cervical rotation-traction manipulation makes a forward traction in preparation for the thrust while reducing the thrust amplitude. In addition, the pre-traction of cervical spinal manipulation can enlarge the sagittal plane of the intervertebral foramen, reduce the internal pressure of the nucleus pulposus, help to avoid the secondary damage to the intervertebral disk caused by the pure rotational force, help to release the adhesions of the ligaments around the surrounding small joints, increase the mobility of the intervertebral joints, narrow the range of motion of the cervical spine during the thrust, and facilitate the safe operation of the manipulation (Li et al., 1998; Anderst et al., 2018). Through the photos we found that the cervical rotation-traction manipulation has a larger body contact area than the oblique pulling manipulation. We believe that the larger body contact area is to transmit the force to the hand through the torso, which can control the force better and the force emitted is more stable. Thereby its thrust amplitude is smaller.

### The Hazards of Large Thrust Amplitudes

Cervical rotation manipulations produce thrusts that move the cervical spine out of its normal physiological range of motion without exceeding the limits of anatomical integrity (Chaibi and Benth, 2017). Klein et al. (2003) used a three-dimensional space-measuring instrument to measure motion parameters during the cervical spine rotation manipulation. They measured the three-dimensional range and amplitude of motion of the left and right cervical segments (C3 and C5). They found the maximum amplitude between the head and trunk during thrust did not exceed the physiological range of activity. Passmore et al. (Chaibi and Benth, 2017) used a cervical range-of-motion goniometer to measure improvement in mobility after cervical spine manipulation. They concluded that cervical rotation to the right resulted in a significant improvement in range of motion of 3.75 degrees.

However, at the same time, torsion is the most significant risk factor for disk injury, especially in the pathological state of the disk (Harvey-Burgess and Gregory, 2019). The intervertebral disk is the most critical part of the cervical spine load-bearing system. Torsional loading produces shear stresses in the horizontal and vertical planes of the disk, which are proportional to the distance between the axes of rotation. Biomechanical experiments (Schmidt et al., 2007; Veres et al., 2010) have shown that when the spine is flexed and the compound torque is rotated, the disk is subjected to large shear forces, and the compound motion of repeated flexion plus rotation may damage the disk. At this point, excessive rotation may lead to disk herniation or even prolapse. The intervertebral disk is viscoelastic tissue (González Martínez et al., 2017) that undergoes elastic changes in a physiological state after compression and is linear. Once subjected to larger shear forces or repeated excessive stresses it becomes non-linear which is a plastic change. It inhibits the synthesis of the disk matrix and decreases its content, leading to an increased risk of disk degeneration. While moderate stress is essential to maintain normal disk nutrition, an abnormally high stress environment is an important factor in disk degeneration, which can alter the surrounding environment of disk chondrocytes.

On the other hand, when the Luschka joint of the C3 to C4 vertebrae is in the coronal position, too much thrust force at this time can cause abnormal shear forces resulting in hook vertebral fractures. In addition, excessive thrust amplitude may cause cervical dislocation, small joint displacement, cervical instability and intervertebral joint disorder. Cervical spine manipulation is widely used in relieving cervical myofascial pain and increasing cervical spine mobility. In this experiment, we chose volunteers who were healthy and cervical spine manipulation was relatively safe to operate in normal subjects. However, when cervical spine manipulation is used to treat patients with cervical spondylotic radiculopathy, the wide range of rotation during retraction may aggravate cervical disk herniation and compress the nerve roots and cervical spinal cord.

## Limitation

However, this study has some limitations. First of all, this study involved only one clinician, and the mechanical parameters of CRM may vary considerably between practitioners of different genders, sizes, and clinical experience. Secondly, the subjects were young, healthy individuals, so the results of the study may not be generalizable to other populations. Thirdly, only two subcategories of non-fixed-point rotational manipulation were explored in this study, and the mechanical parameters of non-fixed-point rotational manipulation cannot be directly extrapolated to fixed-point rotational manipulation. In order to further investigate the clinical efficacy and safety of CRM for cervical radiculopathy, the next step will be to select other techniques, such as fixed-point rotation, and recruit patients with varying degrees of cervical spondylosis as volunteers for basic research as well as practitioners with different gender, age and other influencing factors. Questions such as whether the kinematic characteristics of twisting and lifting techniques with smaller thrust amplitude displacements are regular, and whether they are common to different clincians in different subjects need further refinement.

## Conclusion

In summary, the mechanical parameters and active and passive motion amplitudes of the oblique pulling manipulation are similar to those of the cervical rotational traction manipulation. However, in terms of thrust amplitude, the oblique pulling manipulation has a greater amplitude and therefore maybe poses a greater risk of potential cervical spine injury during manipulation than the cervical rotational traction manipulation.

## Data Availability Statement

The raw data supporting the conclusions of this article will be made available by the authors, without undue reservation.

## Ethics Statement

The studies involving human participants were reviewed and approved by Research Ethics Association, Shunde Hospital Affiliated to Guangzhou Medical University. The patients/participants provided their written informed consent to participate in this study. Written informed consent was obtained from the individual(s) for the publication of any potentially identifiable images or data included in this article.

## Author Contributions

DL completed the experiment and wrote the manuscript. XH directed the design test and repaired manuscript. The rest of the authors were put forward valuable opinions in the whole subject design. All authors contributed to the article and approved the submitted version.

## Conflict of Interest

The authors declare that the research was conducted in the absence of any commercial or financial relationships that could be construed as a potential conflict of interest.

## Publisher’s Note

All claims expressed in this article are solely those of the authors and do not necessarily represent those of their affiliated organizations, or those of the publisher, the editors and the reviewers. Any product that may be evaluated in this article, or claim that may be made by its manufacturer, is not guaranteed or endorsed by the publisher.
